# Indicators of social capital in prison: a systematic review

**DOI:** 10.1186/s40352-015-0020-8

**Published:** 2015-04-11

**Authors:** Lise Lafferty, Georgina M Chambers, Jill Guthrie, Tony Butler

**Affiliations:** 1 The Kirby Institute, University of New South Wales, Kensington, 2052 Australia; 2 National Perinatal Epidemiology and Statistics Unit, University of New South Wales, Kensington, 2052 Australia; 3 National Centre for Indigenous Studies, Australian National University, Canberra, 0200 Australia

**Keywords:** Social capital, Social support, Prisoners, Incarceration, Prison

## Abstract

**Background:**

Social capital theory encapsulates multidisciplinary principles and is measured across numerous social entities. However, there is a paucity of literature exploring the benefits of social capital for sentenced prisoners.

**Methods:**

A qualitative systematic review was conducted using the PRISMA Guidelines. Eight databases were searched; thirty-one articles met the inclusion criteria.

**Results:**

Social capital was found to exist across a number of themes/dimensions for sentenced prisoners. The benefits gained were sometimes gender-specific and differed between social capital available in prison and that provided externally.

**Conclusion:**

Social capital is a valuable resource among prisoners and has the capacity to improve quality of life outcomes. Further research exploring the causality of social capital and improved outcomes among prisoners is needed.

## Background

Social capital is a relatively recent concept encompassing social support, social networks and social cohesion (Almedom 2005). Social capital is reliant on participation of more than one person and allows for individuals to utilise resources which they would not otherwise be able to access if acting independently. Social capital can be used to improve a person or community’s quality of life, including improved health and wellbeing.

Social capital theory has been developed across a number of disciplines including social science, economics, health, public policy and governance (Shortt 2004). Although there is a discrepancy about the term social capital, there is widespread cross-discipline agreement that “there is a social effect being measured” (Scheffler and Brown 2008:324). Social capital has been measured across numerous social entities including the home/family, community, and at city, regional and national level (Krishna and Shrader 1999).

Instruments used to measure social capital are as diverse as the contexts in which this ‘public good’ is measured. While there exist a number of constructs and instruments to identify and measure social capital in the general population, there is a paucity of literature which explores social capital in marginalised groups, including within the prison context.

By exploring the various definitions available, it is apparent that social support is a common theme within the social capital literature as being a fundamental ingredient to the development of social capital. Social support is often measured through relationships and reciprocity, evidencing a person or group’s capacity to call on others.

Both social capital and social support exist across a number of realms and there is not a single measure of social capital or social support or a single focus. Social capital and social support are often discussed and measured in combination with other themes, such as in the context of social capital and religion (Saguaro Seminar on Civic Engagement 2000), social capital and education (Coleman 1988), and social capital and health (Carpiano 2007; Giordano et al. 2011).

Although social capital is often considered a social resource positively enabling groups or individuals to benefit, social capital can render negative outcomes. To use Putnam’s (2000) example, it was the negative consequences of social capital that enabled the perpetrator of the widespread destruction and fatalities in the Oklahoma City bombing. Portes (1998) identified four potential negative outcomes of social capital. These are: exclusion of others (such as ethnic groups that enable the development of social capital which, invariably, occurs at the social exclusion of those from other ethnic groups); insular obstruction (whereby entrepreneurial endeavours are prevented from expanding); conformity (the requirement to conform when groups function as a singular entity); and resistance (when adversity or oppression is the common bond within a group – individual successes are often not supported by the wider group) (Portes 1998).

This systematic review explores the information that is known about social capital and social support available to incarcerated adults and identifies gaps in the literature relating to the social capital of this population. This review reports on the social capital mechanisms accessible by inmates and how these mechanisms enhance an inmate’s outcomes and quality of life. It seeks to identify the instruments which currently exist to measure social capital among incarcerated inmates and the dimensions of social capital measured.

Social capital has been shown to improve quality of life through greater access to resources that may not otherwise be available to an individual acting alone such as health outcomes (e.g. smoking cessation (Rocco and d'Hombres 2014)) and educational attainment (Coleman 1988). Social capital is often defined by geographic or contextual proximities such as neighbourhoods or groups (i.e., community organisations and self-help groups such as Alcoholics Anonymous). Thus, it might reasonably be expected that outcomes related to social capital outside of prison would also exist within prison. However, we would argue that due to the unique social and cultural milieu within a prison environment, identifying social capital within that environment requires alternative measures and constructs from those used to measure social capital *in the community*.

## Methods

This review was conducted through database searches and was completed using the PRISMA statement – a guideline for “transparent and complete reporting of systematic reviews and meta-analyses” (Liberati et al. 2009).

Articles were assessed as eligible for inclusion if they related to adults aged 18+ (as this is the common minimum age for incarceration within adult correctional centres internationally and filters out juvenile offenders who may be experiencing different social support needs), sentenced prisoners (i.e. not on remand, parole, or probation), and inclusive of social capital and/or social support. Sentenced prisoners were chosen to streamline the incarceration experiences as prisoners held on remand may have different social and emotional needs related to the uncertainty of their incarceration. Additionally, prisoners held on remand often do not have access to employment, certain health treatments, education and social programs in prison.

Although social support has not been used as a search term in other systematic reviews looking at social capital within a particular population group (such as mental health, see De Silva et al. 2005), the term was not singularly used to search databases within this review as the term ‘social capital’ produced minimal results. Using both ‘social capital’ and ‘social support’ as a search term, combined with ‘prisoner’ or variations of this word (e.g., ‘inmate’ or ‘offender’), broadened the search to include thematic diversity among the studies and provide greater insight into not only the social dynamics and constructs prevalent among inmates and within prison cultures but also the social resources that exist within these environments.

The inclusion of social support as a search term assisted in creating a more exhaustive search of the existing literature relating, contextually, to social capital. Studies that focused on prisoner re-entry, prisoners of war, those under house arrest, the incarceration of children, adolescents and juveniles were excluded. Publications relating to social support for incarcerated parents were included, but publications focusing on the children of incarcerated parents were excluded. The following databases were searched: CINAHL, Informit, EMBASE, PubMed, Web of Science, ProQuest, PsycINFO, and Scopus. Search terms included: prisoners, criminals, offenders, inmates, social capital, social support, and social support index. The studies were searched from as early as possible until 9^th^ October 2013. Some databases had pre-set search dates dependent on the search term entered. For example, the search term “prisoner” resulted in a pre-set search date from 1981-2013; in this same database, the combined search term ““prisoners” + “social capital”” resulted in a pre-set search date from 2002-2012. Search Term Combination ‘”social support” AND prisoner’ (or ‘offender’ or ‘inmate’) were then used.

**Figure 1:**
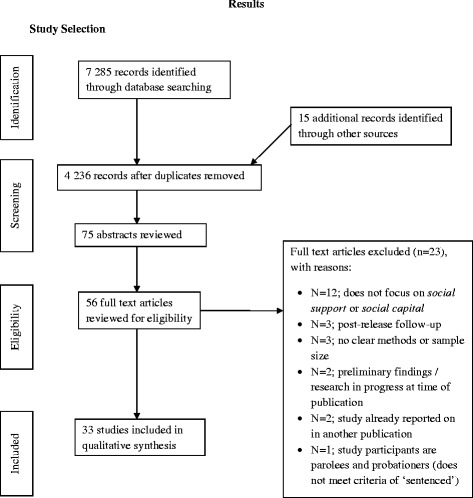
**PRISMA flow diagram of study selection.**

The search resulted in 7300 records (n = 7285 identified through databases and n = 16 identified through other sources). Duplicate titles were removed. The titles of the literature were reviewed and subsequent abstracts reviewed. Papers were read (n = 56) for final inclusion (n = 33)/exclusion (n = 23) (Figure 1).

## Results

### Synthesis of results

The following thematic categories were identified from a review of the literature: Relationships (n = 9), Religion (n = 6), Addiction (n = 4), Prison Climate (n = 3), Visitation (n = 3), Ageing (n = 3), Self-Harm & Suicide (n = 2), Gang Affiliation (n = 2), Mental Wellness (n = 1), and Civic Engagement (n = 1). One publication had dual categories (relationships and visitation), resulting in more category records than total number of publications included in the systematic review.

## Discussion

### Relationships

Intimate relationships in prison are often formed through power and control, with one partner being exploited either sexually, or economically, or both. Beer *et al* (2007) conducted a quantitative comparison study to explore the impacts of marital and relationship status on well-being, including anger and prison adjustment, among female inmates. Another study pertaining to intimacy and women in custody, conducted by Greer (2000), used qualitative analysis to reflect on the nature of relationships within a female prison from friendships to sexual intimacy, commonly based on sexual exploitation rather than a genuine relationship.

Beer, *et al* (2007) utilised two instruments capturing measures of social capital in their research: the Social Support Questionnaire – Six Item Brief Measure – Revised (SSQ-6-R) and the Relationship Assessment Scale. Beer *et al* (2007) found that women in relationships, whether with someone outside the prison or someone inside, often experienced greater levels of anger and hostility as well as greater challenges with adjustment to prison, manifesting in behaviours and actions resulting in disciplinary infractions. Intimate relationships are commonly understood to provide a source of social support within the general population. However, heterosexual women in prison who engage in same-sex relations may experience high levels of distress in conjunction with any supports or capital they ascertain from the relationship due to the inner conflict experienced by heterosexual women in same-sex relationships. Both Beer *et al* (2007) and Greer (2000) reported perceived self-serving motivations (e.g. economic gain) were found to be the primary reason for ‘attraction’ among many same-sex relationships in prison.

Larson and Nelson (1984) and Desmond (1991) explored the impact of friendships among women prisoners in the United States. Friendships were examined as a key adaptation variable (Larson and Nelson 1984). Those who had several friends and prior incarceration experience had lower levels of “perceived powerlessness” (Larson and Nelson 1984), suggesting that social networks, or possibly social capital, have a buffering effect for women adapting to prison life. However, Larson and Nelson (1984) note that substantial time remaining in an inmate’s sentence fostered high levels of powerlessness. Women perceived themselves as “efficacious in prison life” if they retained positive bonds with friends and family who were on the outside (Larson and Nelson 1984:607). Women with negative expectations about their return to community, coupled with intense friendships in prison and contrasting feelings of solidary were found to have “a more salient criminal identity” (Larson and Nelson 1984:610). Isolation from friends and family on the outside can result in negativity towards the law. Women who lack social capital both inside and outside prison may experience social deficit on their return to life on the outside which may exacerbate their perceived dire or hopeless situation and perpetuate the cycle of incarceration.

Loneliness among women prisoners can be detrimental to a woman’s prison adjustment and her criminal identity. However, in a study which sought to identify if a relationship existed between social interaction and loneliness among female inmate, Desmond (1991) found that loneliness scores were the same between those whom and without someone on the inside with whom they could confide. One of the instruments used in Desmond’s study was the Revised University of California Los Angeles Loneliness Scale (UCLALS), a measure of loneliness relevant to relationships with others (Desmond 1991).

Surrogate relationships, where women seek out other inmates to fulfil familial absences, were discussed by Severance (2005) and Loper & Gildea (2004). Surrogate relationships may fulfil familial absences while in prison in an attempt to find comfort or support, such as a mother, a sister, or an aunt would provide on the outside. However, surrogate relationships can have both positive and negative consequences.

Severance (2005) observed that relationships were broadly categorised into four categories: acquaintances, friends, family, and girlfriends. “Few inmates specifically discussed pseudokinship roles and functions. In general, comments about pseudofamily relationships were not favourable” (Severance 2005:357). Family relationships were reported with less frequency than ‘friends’ or associates’ (Severance 2005).

Younger women were more likely to have a higher number of surrogate relationships and tended to benefit from greater levels of support from their surrogate families than did older inmates (Loper and Gildea 2004). This may be a consequence of younger inmates’ vulnerability and they may seek out surrogate families for protection and as a buffer against isolation (Loper and Gildea 2004). An instrument that measures aspects of social capital, the Prison Personal Support Questionnaire (PPSQ), was designed by Loper and Gildea to capture the social connections of incarcerated people in the study (Loper and Gildea 2004).

Men who “enjoyed strong and consistent family support”, either through family visits, letters, and financial assistance, maintained “a high level of self-esteem” (Leahy 1998:284). Leahy (1998) describes this group’s most distinguishing characteristic was that of inner motivation. Based on this inner motivation/drive, coupled with external supports, these men appear to have had greater access to social capital than did those who viewed themselves as ‘beyond rehabilitation’.

Among men, social support scores were comparable for families and significant others whilst social support from friends scored lower (Swanson et al. 2012). This suggests that inmates in this study valued the social support of their significant others and families above that of friends. Findings showed a positive correlation between social support from friends (both inside and outside of prison) and the longer an inmate was incarcerated (Swanson et al. 2012).

The juxtaposition of educational level and its influence on perceptions of social support may provide insight into how education levels are internalised. Inmates with a high school diploma or GED (a high school equivalency certificate) perceived their social support from friends, family and significant others to be the highest. Inmates with education levels below a high school diploma/GED or, conversely, above, such as a college degree, perceived lower levels of social support from friends, family, and significant others (Swanson et al. 2012). However, inmates with college degrees may be experiencing status/identity issues as they find themselves within prison or, given their social status within society as educated individuals, they may have been abandoned by their social support networks during their conviction.

Participation in programs, such as education and rehabilitation (e.g. Alcoholics Anonymous), in prison was correlated with perceived social support from significant others (Swanson et al. 2012). The authors attribute this variant to these programs providing access to potential mentors whereby bonds and trust may be developed – key ingredients in the investment of social capital.

Married inmates reported higher levels of depression and anxiety. Inmates with close relationships inside of the jail reported higher levels of hostility, although gender differences exist – women were clearly affected by these traits whereas men did not display these characteristics (Lindquist 2000). A similar pattern was evident in Beer *et al*’s (2007) research whereby incarcerated women in a relationship with someone inside or outside the prison experienced increased hostility and anger. Nearly three quarters of inmates ’disagreed‘ or ’strongly disagreed‘ that family and friends would stick by them (Lindquist 2000). Marital status was found to be the most significant predictor when looking at the “impact of social relationships on anxiety” (Lindquist 2000:445).

### Religion

Religion is commonly considered to be a dimension of social capital as it can be a (re)source to empower individuals to improve their lives (Putnam 2000). Religious participation is considered a source of social capital (Saguaro Seminar on Civic Engagement 2000) with religious participation regarded as evidence of group membership and bonding through the shared interest among the group’s members (Putnam 2000).

One study which attempted to quantify male prisoners’ religiosity found that those reporting higher levels of religiosity had fewer disciplinary confinements than those reporting lower levels of religiosity (Clear and Sumter 2002). The Rosenberg Self Esteem Scale (RSE) was used in this study to capture an individual’s agency, or their global self-esteem. Those reporting higher levels of religiosity experienced better adjustment to prison than those reporting lower levels of religiosity. Depression was found to play a role in inmate adjustment and religiosity with higher scores on the adjustment questionnaire associated with lower depression scores (Clear and Sumter 2002). Religious inmates reported greater levels of both self-mastery and self-esteem and lower levels of depression than those who were not religious (Clear and Sumter 2002).

In a study conducted by Kerley & Copes (2009) in a men’s state penitentiary in Mississippi, prisoners who experienced ’religious epiphanies’ in prison found support structures through engagement with like-minded faith followers whilst incarcerated. The shift in social circles following their religious epiphany resulted in the development of positive relationships, both in prison and outside (Kerley and Copes 2009). Those experiencing religious epiphanies asserted “what helped them survive prison life were the social support networks” (Kerley and Copes 2009:236). In another study conducted in the same penitentiary, Kerley, Matthews, and Blanchard (2005) found that believing in a higher power provided a 70% reduction in the likelihood of frequent arguments in prison.

In Liebling & Arnold’s study of an English men’s prison, conversion to Islam appeared to be a facade for gang initiation. As one participant commented, “*Violence is currency in prison*” (Liebling and Arnold 2012:413). In this context, religiosity is a form of negative social capital, however, affiliation was found to be necessary for survival in prison.

Prisoners were found to align themselves with other “self-protecting prisoners” (Liebling and Arnold 2012:416). Liebling and Arnold’s (2012:418-420) results support the “hypothesis that Muslim prisoners felt more ‘collective’ as well as ‘oppositional’ or ‘distrusting of staff’” and “The [Muslim] group were able to capitalise on feelings of fear, hopelessness or loneliness to make people join in”. The vulnerability experienced by new arrivals was also exploited by the Islamisation of prisoners. Within the social capital framework, group membership enables individuals to gain through the contributions of the collective. This conversion to Islam was structured in a way which provides individual benefit to its members through security and protection.

Camp *et al*. (2006) explored the types of inmates, including men and women, who volunteered for participation in an 18-month residential, faith-based program. Camp *et al* (2006) reported inmates who scored higher on the Prochaska-DiClemente Motivation Scale were more likely to volunteer to participate in a faith-based residential program than those with lower scores. Participants scoring higher were more motivated to make changes in their lives than those not participating in the program (Camp et al. 2006). The faith-based residential program participation study also found no relationship between “self-worth or desire for community integration and participation” in the program (Camp et al. 2006:542). This study suggests that engaging with and accessing social capital reflects on a person’s own internal motivators driving them to participate. However, the study does not provide explanations of causation – are a person’s internal motivators the drive for engaging in the program or, does participation result in increased motivation?

Levitt & Loper (2009) examined 213 women who participated in religious activities in prison and level of support (No, Low, Moderate, or High) obtained from their spiritual activities and other activities in prison. Inmates who had been incarcerated for longer (mean = 61.85 months), reported less participation in religious activity than those who had served fewer months (Levitt and Loper 2009). The authors concluded that new prisoners are unaccustomed to the deprivation of prison life and are thus more likely to seek ‘respite in the chapel’ and as inmates become more experienced they form other supportive relationships that replace the support provided through religious activity (Levitt and Loper 2009).

### Addiction

El-Bassel, Gilbert, Schilling, Ivanoff, and Borne (1996:43) conducted a study among female inmates to determine if “women who report childhood and adult psychological traumas are more likely than other women to be classified as regular crack users”. This research used the Inventory of Social Supportive Behaviours (ISSB) (a measure of tangible and intangible social support received during the preceding month) and found that women who perceived less emotional support were more likely to be regular crack-cocaine users, while those women who indicated higher emotional support were less likely to be crack-cocaine users. Other studies found women were more successful in drug abstinence when they perceived being supported in their efforts (Bock et al. 2013; Chen 2010).

Bock *et al*. (2013) explored social support factors which might influence female inmates’ ability to remain smoke-free following release from prison. Among other scales relevant to the study, the authors utilised the Interpersonal Support Evaluation List (ISEL) – an instrument which measures social capital through its measuring of a person’s interpersonal support. Smoking outcomes were significantly correlated with confidence, motivation, readiness and plans for post-release smoking abstinence (Bock et al. 2013). However, the authors noted that temptations to smoke appeared more related to family than friends (Bock et al. 2013); a finding corresponding with Swanson *et al*. (2012) research which found that social support from family was valued above that of friends.

Over half of the participants thought family members would encourage their decision to remain smoke-free but less than one-third thought their friends would encourage this (Bock et al. 2013). It can be speculated that persons who *perceive* higher levels of social support are more likely to be successful in making lifestyle changes such as quitting tobacco or drugs that positively impact on their health.

Participants in a study conducted with inmates who were abstinent from substances and accommodated in drug-free wards at state penitentiaries in Israel were categorised into two groups: (1) those who had been drug abstinent more than one year; and (2) those who had been drug abstinent up to one year (Chen 2010). Women who were drug-abstinent for one year or more had significantly more friend support than women having abstained less than one year (Chen 2010). A similar contrast was not found in the male sample. A gendered variance in the results showed that men in prison experienced lower perceived family support (rather than friend support) and that a lower sense of coherence predicted higher level trait anxiety (Chen 2010). However, for female inmates, this predictor occurred similarly but with lower perceived friend support (rather than family) (Chen 2010). The findings of the study provide support for the hypothesis that gender differences exist “in the sense of coherence, perceived social support, and negative emotions among drug-abstinent inmates” (Chen 2010:951). This is an important finding evidencing gender differences exist within the construct of social capital.

Staton-Tindall, Royse, and Leukfeld (2007:238) examined “the extent to which substance use and criminality influence perceptions of social support among incarcerated women”. The authors used the Multidimensional Scale of Perceived Social Support, a 12-item scale capturing the social support elements of social capital, and the Addiction Severity Index in their research. Lower perceived social support and smaller social network sizes were found to be predicted by more severe use of alcohol and drugs (Staton-Tindall et al. 2007).

### Prison climate

An exploration into the social climate of Australian prisons utilised the prison social climate measure, EssenCES, to measure “key aspects of a social climate that are considered relevant to offender rehabilitation” (Day et al. 2011:5). Results showed that prisoners felt safer than both clinical and operational staff in the prison environment (Day et al. 2011). Within the context of safety as a dimension of social capital, this may be reflective of aspects of social capital resources existent for inmates in custody but lacking in availability for operational or clinical staff. The authors report that “more positive perceptions of the social climate were associated with higher levels of readiness for treatment” (Day et al. 2011:4) among the study sample. Higher staff morale and reduced stress among the study sample were found when the social climate was perceived as a more positive environment (Day et al. 2011).

While the EssenCES measure does not directly capture social capital, it measures elements of social capital such as safety, inmates’ social cohesion and mutual support (whereby the care expressed between inmates is measured), and hold and support (a measure of the level of support provided by staff to inmates).

A paper on differential coercion and social support within the prison environment evidenced that social capital can be developed between inmates and prison officers in a capacity reliant on mutual trust and reciprocity. From 1968-1972, inmates at the Penitentiary of New Mexico were actively engaged in opportunities stemming from civic engagement within the prison environment (Colvin 2007). This self-advocacy extended to include the establishment of direct relationships between inmates and external education providers and volunteer services, as inmates sought to build their educational and vocational capacities whilst completing their sentence. However, as the paper demonstrates, shifts in governance and funding led to a social capital shift within the prison environment; a turn of events resulting in rapid descent from positive social capital, in which inmates were able to pursue higher education, to negative social capital, in which drugs and other illicit economic activities emerged, ultimately resulting in one of the worst prison riots in United States history (Colvin 2007). From this case study, it is apparent that the social support provided by guards to inmates and attributable to positive social capital in the prison environment can, when a shift in power digresses from mutual contributions to one of coercion, have detrimental effects.

Another study exploring the relationship between prisoners and guards, in Israeli prisons, found that social support and reciprocity can exist between the two groups, despite the contextual power dichotomy that fundamentally exists within the prison environment (Shapira and Navon 1985). In this study, conducted by Shapira and Navon (1985), findings showed that the supply of contraband to prisoners from the guards, resulted in greater cohesion between the groups. This suggests that transactions which appear to represent negative social capital can, in some circumstances, be an indication of positive social capital as groups work collaboratively towards a common goal of social cohesion.

### Visitation

Cochran (2012) explored the frequency of misconduct and patterns of prison visits for male and female inmates in Florida prisons. Four visitation categories were identified: non (never visited), early (visited more frequently in the early months of incarceration), late (visited more frequently in the later months of incarceration), and consistently visited; and three misconduct categories: no, low, and high. Twenty-eight percent of inmates who never received visitors were in the ‘low misconduct’ category, while only 21-23% of inmates in the three groups who received visitors were in the ‘low misconduct’ category (Cochran 2012). The early visited inmates were more likely to fall into the ‘high misconduct’ trajectory than any of the other groups (Cochran 2012). The author speculates that “the findings here lend further support to the notion that visitation reduces the likelihood of inmate misconduct” (Cochran 2012:438).

Desmond (1991) considered whether receiving visitors counters a female prisoner’s experiences of loneliness whilst incarcerated but the results did not support the hypothesis.

Wooldredge (1999:243) examined a number of dimensions of social capital including group participation (as measured through “the number of hours spent daily in structured activities”), connection / engagement with the outside world (as measured through “frequency of visitation with outsiders”), and safety (as measured through “whether an inmate was victimised recently by physical assault”) among male inmates at two maximum and one medium security facilities in Ohio. Prisoners who engaged in fewer hours of structured activities, received fewer visitors, and were recent victims of aggravated assault were found to experience greater levels of depression, anxiety, stress and other indicators of decreased psychological wellbeing (Wooldredge 1999). These results indicate that greater isolation potentially translates into lower levels of social capital.

### Ageing

While ageing itself is not a dimension of social capital, the social and physical attributes of ageing may both interact and interfere with our capacity to engage in activities capable of developing or maintaining social capital reserves.

Gallagher (1990) published a comparative study relating to the social network characteristics and the physical and emotional health of older and younger male inmates. This study used visitation and other contact / correspondence (such as phone calls and mail) with friends and family on the outside as an indicator of social support resources available to inmates (Gallagher 1990). Such indicators are highly relevant to identifying and measuring an inmate’s social capital and are consistent indicators across many social capital measures. Older inmates were more likely to receive visitors, have more friends in prison, and have a confidant inside prison compared with younger inmates (Gallagher 1990). Older men were more likely than younger men to confide in a staff member of the prison whilst younger men were more likely to place trust in a fellow inmate. Gallagher (1990) suggests this may reflect the similarity in ages of the older inmates with staff and perhaps shared experiences.

Krabill & Aday (2007) conducted a study on the utilisation of perceived social support and shared social interactions in the development of supportive social networks among ageing women in prison. Eighty-six percent of participants reported feeling emotionally close to relatives and could rely on family for social support (Krabill and Aday 2007). Letter writing was reported as the most common method of communication with loved ones on the outside, followed by phone calls and in-person visitation (Krabill and Aday 2007). Communication and correspondence with loved ones on the outside is influential to an inmate’s perceived level of social support available.

Participants were more inclined to provide support to other inmates who were unwell (Krabill and Aday 2007). Both age and proximity (elements indicative of bonding social capital) were shown to be factors for prisoners when selecting friends inside, with roommates the most likely person participants confided in (Krabill and Aday 2007). This is one example in which social capital among incarcerated women can provide healthful gains. The authors reported that “the prison not only confines prisoners, but also provides the impetus to manufacture new social networks as a prison strategy” (Krabill and Aday 2007:49).

A comparative study inclusive of male and female inmates compared with non-inmates found a correlation between network size and age, with a reduction in network size as age increased (Bond et al. 2005). This study implemented the Social Convoy Questionnaire (modified) as a means of mapping the social networks of study participants. Older inmates had closer networks with less peripheral excess than did their younger counterparts, effectively creating a social bubble for themselves within the prison. The authors noted that “smaller, very close social networks seem to increase in emotional value to the older inmate […], perhaps by personal choice or perhaps through ageism, older inmates might interact within this small buffered network experiencing some semblance of emotional closeness, even in their harsh context” (Bond et al. 2005:174-5).

### Self-harm & suicide

Rivlin, Fazel, Marzano, & Hawton (2012) utilised a number of instruments including the Social Support Scale (SSS) (an instrument which measures elements of social capital) in a study which explored suicide behaviours among male inmates from 19 prisons in England. This study compared prisoners who “made near-lethal suicide attempts in prison” with “prisoners who had not engaged in near-lethal self-harm in custody” (Rivlin et al. 2012:2). Employment, although generally attributed to human capital rather than social capital, was found to be more prominent among those who had not attempted near-lethal suicide in custody (Rivlin et al. 2012). Those who were employed whilst incarcerated were less likely to attempt suicide, thus suggesting the value of employment in an assessment of a person’s access to social capital (Rivlin et al. 2012).

Social support was correlated with attempted suicide – those who had lower levels of social support were more likely to make an attempt on their own life (Rivlin et al. 2012). In regards to social networks, Rivlin *et al* (2012:4) reported those making near-lethal suicide attempts were more likely to report “none or few close or good friends outside prison”. However, having more “close or good friends living or working inside prison” than those who had not attempted suicide. In other words, friendships on the inside are not a protective factor against suicide attempts. This finding reflects that an inmate’s social capital is enhanced through social support and connection with the outside world. These connections may be significant for prisoners in maintaining quality of life whilst incarcerated.

A study conducted in Western Australia explored male inmates’ likelihood to approach prison officers for support including both emotional support and practical assistance. A participants’ likelihood of seeking support was examined within the context of history of self-harm (Hobbs and Dear 2000). There was no statistical significance for inmates in seeking assistance between those with a history of self-harm and those without (Hobbs and Dear 2000). The authors note that “if prisoners’ only access to support is through prison officers, then prisoners in need would be reluctant to seek help” (Hobbs and Dear 2000:127). This observation indicates a lack of social capital provided by officers within the prison environment – particularly among a vulnerable group of inmates.

### Gang affiliation

A longitudinal study conducted by Mears, Stewart, Siennick, & Simons (2013:695-6) found that “the code of the street belief system affects inmate violence and that the effect is more pronounced among inmates who lack family support, experience disciplinary sanctions, and are gang involved”. This study, consisting of both male and female participants, provides insight into the significance of a lack of positive social capital (through family support) combined with negative social capital (through gang involvement). The stronger an inmate’s belief in the street code, the more likely the inmate was to engage in violence while incarcerated (Mears et al. 2013). Adherence to the street code is different than the inmate code – the street code is imported from pre-incarceration into the prison environment and, as the authors report, clearly affects inmate violence within the prison.

Mears *et al* (2013) indicate that a number of factors contribute to inmate violence including gang involvement (this was evidenced by Gaes *et al* (2002)), lack of family support, and disciplinary actions. In terms of social capital, it can be speculated that bonding social capital – attained through group membership such as educational and religious participation – may positively influence inmates whereas a lack of family support, prisoner maladjustment and gang affiliation may negatively influence inmates. Increased adherence to the street code coupled with these negative indicators are more influential on an inmate’s disposition to commit violent acts above the inmate’s belief or commitment to the street code (Mears et al. 2013).

In a study exploring gang membership on violence among male inmates and identifying a “threat index”, the amount of time spent in a gang was negatively correlated with an inmate’s violent misconduct – i.e., the longer an inmate spent in a gang, the lower their likelihood of committing violence (Gaes et al. 2002). However, it is possible that ageing inmates may gain authority with age and delegate younger inmates to commit violence. “Core members” of gangs were more likely to commit violent misconduct than were “peripheral members” (Gaes et al. 2002:381). This suggests that the greater invested a person is within the gang, the greater the negative social capital produced from their membership/participation.

### Mental wellness

Gender differences in social support and mental well-being among incarcerated adults were explored in a study conducted by Hart (1995). This study used two instruments which capture social capital: the Inventory of Social Supportive Behaviours (ISSB) (a measure of tangible and intangible social support received during the preceding month) and the Rosenberg Self Esteem Scale (RSE) (a measure of an individual’s agency) (Hart 1995). Four sources of support were identified: 1) Emotional support: “where individuals are accepted or esteemed”; 2) Informational support: “are aided in understanding and coping with problems”; 3) Social companionship: “spending time with others”; and 4) Instrumental support: “the provision of material aid and services” (Hart 1995:68). Collectively, these articulate the means of social capital. Hart (1995) found that women prisoners had higher levels of social support than men. The study found a “significant relationship between social support and psychological well-being, specifically self-esteem, for female inmates”; a similar relationship was not evident for male inmates (Hart 1995:85). However, Beer *et al*. (2007) and Lindquist (2000) both found greater levels of anger and hostility among female inmates.

### Civic engagement

The only study to consider an inmate’s civic engagement, as expressed through voting (and their ongoing right to), was by Behan (2012). Behan examined the voting experience of Ireland’s incarcerated men in the 2007 national election (legislation was passed in 2006 enabling inmates to vote by postal ballot). This study is of particular interest to the notion of social capital as civic engagement, specifically through the act of ‘casting a ballot’, is widely regarded as a key feature of social capital (Putnam 2000) in mainstream society. In stark contrast, the prison environment is renowned for its oppressive tactics and removal of liberties, thus voting whilst incarcerated is quite unique.

Despite participants reporting a civic duty to vote, a number of responses explaining abstention from voting indicated a lack of social capital (Behan 2012). These ranged from a belief that the new government would do little to change policy impacting on prisoners and a “lack of trust in politicians and alienation from civic society” (Behan 2012:21). The requirement of providing an address for registration was off-putting for many – some inmates cited homelessness (before incarceration) while others simply did not want to list the prison. Behan notes that many inmates reported feeling neglected by the world beyond the prison’s walls, commenting that some prisoners “refused to vote because they felt the outside world had no bearing on prisoners, individually or collectively” (Behan 2012:23).

These findings say much about the potential for reintegration following release from custody for some inmates – whether successful or unsuccessful. Civic engagement, i.e. voting, as expressed by the inmates within this study, is a critical feature of social capital as it describes and expresses a connection beyond the self – active participation in the way the world around the individual is governed and operates. Civic engagement may be a stepping stone towards community involvement; it may continue to foster ongoing involvement rather than withdrawal.

### Limitations of the studies

The studies described above note a number of limitations including those influenced by study design and unforeseeable events or circumstances within a prison study context. Most studies acknowledged that results were not generalisable to other inmate populations, for various reasons including gender and cultural variances, selection bias, self-reporting bias and recall bias. As a number of studies were not longitudinal, and many faced institutional barriers to entry and data collection, causation could often not be determined. Self-completion surveys may result in limited validity due to the lower literacy rates of inmates compared with that of the general population. With regards to recall bias, participants may have provided answers that were favourable to the researcher or did not compromise the inmate’s status or opportunity for release (i.e., inmates may not want to disclose their drug use whilst incarcerated if they will be up for release soon). Another limitation within the literature is a seeming inability to define or identify the direction of causality. A number of studies highlight implications relating to high or low levels of social support/social capital, but were unable to identify if these implications were a result of the level of social capital or if the level of social capital was a result of the implication. Were inmates with low social capital prone to higher infractions or were their behaviours limiting access to social capital?

### Limitations of the review

The authors of this review provided a thematic framework of the literature within the Discussion. However, there are overlapping domains and themes with analyses that may have included inspection into the influences of gender, race, culture, or other sociodemographic confounders. Some of these findings have not been reflected within this review as, to do so, would have required meta-analysis of the literature. Furthermore, social capital is a relatively new theory, gaining wider attention within the literature in the 1990s with Putnam’s book *Bowling Alone*. The current conceptual and, as noted above, causative framework, is not yet well articulated. There is limited understanding with which to assess social capital as a causative process as it relates to personal functioning and social interaction.

## Conclusion

The studies included in this review covered broad dimensions of social capital. These included relationships (including intimate/romantic, friendships, family and surrogate), religion, addiction, visitation, ageing, self-harm & suicide, gang affiliation, mental wellness, civic engagement, and prison climate. Studies reported on the association of perceived social support, religious participation, perceptions of societal inclusion/exclusion (as evident in an inmate’s ability to exercise their right to vote), relationships within prison and the perceived or subjective benefits attained through these relationships.

In looking at the literature, a person’s access to social capital is not confined to the interior of the prison. Inmates may draw on sources of social capital both in prison and from outside. Maintaining communication with loved ones on the outside was found to be influential in the levels of perceived social support for women inmates (Krabill and Aday 2007). Swanson *et al*. (2012) found that male inmates experienced higher levels of social support from family than from friends while Leahy (1998) observed that men who received consistent family support were able to maintain high levels of self-esteem. Similarly, Hart (1995) found that social support was significantly linked with self-esteem for women.

Larson & Nelson (1984) found that having several friends (in prison) was a buffer against perceived powerlessness for women with prior incarceration experience while women who maintained closeness with friends and family on the outside were more likely to perceive themselves as being successful on the inside. Women experienced loneliness equally regardless of having someone on the inside or the outside with whom to confide (Desmond 1991). Male inmates who made near-lethal suicide attempts in prison commonly did not have anyone close on the outside but, may have had friends on the inside (Rivlin et al. 2012).

The experiences of relationships inside and outside prison were sometimes gender-specific. Beer *et al*. (2007) and Lindquist (2000) both found that women with friends on the inside reported higher levels of anger and hostility but, men with friends inside did not report similar traits. Rivlin *et al*. (2012) reported that having friends on the inside was not a protective factor against near-lethal suicide attempts among men.

A recurrent theme within the literature was that there are no clear causal pathways for social capital and outcomes in the prison context. There existed a number of correlation findings, but their causality could not be determined. This was particularly evident in Camp *et al*’s (2006) study which found correlation of internal motivation among faith-based program participants.

Social capital among inmates has a multitude of complexities as prisoners are in an environment whereby their personal agency is limited, role of authority is constant, and access to friends and families are monitored. Inmates may collectively participate in activities which collectively contribute to the construction of social capital – both positively (such as through peer-based self-help groups) and negatively (such as the perpetration of violence). A prisoner’s social capital may be enhanced by their connections with the outside world, such as frequent visits and correspondence with loved ones. Equally, inmates may experience greater isolation and a sense of feeling forgotten by their families, communities, and society, resulting in a greater deficit of social resources.

A better understanding of what social capital is within the prison/inmate context is needed. A social capital instrument designed specifically for the prison environment would allow for better exploration of the ways in which social capital influences and interacts with prisoners’ lives in prison.
